# Exploring gut microbiota and its predicted functions in pulmonary tuberculosis: A multi-regional study using public 16S datasets

**DOI:** 10.1371/journal.pone.0336337

**Published:** 2025-11-13

**Authors:** Tejaswini Baral, Anwesh Maile, Nagarajaram Hampapathalu Adimurthy, Kavitha Saravu, Chandrashekar Udyavara Kudru, Jitendra Singh, Chiranjay Mukhopadhyay, Mahadev Rao, Mohan K. Manu, Sonal Sekhar Miraj

**Affiliations:** 1 Department of Pharmacy Practice, Manipal College of Pharmaceutical Sciences, Manipal Academy of Higher Education, Manipal, Karnataka, India; 2 DBT-Centre for Microbial Informatics (DBT-CMI), School of Life Sciences, University of Hyderabad, Hyderabad, Telangana, India; 3 Department of Infectious Diseases, Kasturba Medical College, Manipal, Manipal Academy of Higher Education, Manipal, Karnataka, India; 4 Manipal Center for Infectious Diseases, Prasanna School of Public Health, Manipal Academy of Higher Education, Manipal, Karnataka, India; 5 Department of General Medicine, Kasturba Medical College, Manipal, Manipal Academy of Higher Education, Manipal, Karnataka, India; 6 Department of Translational Medicine, All India Institute of Medical Sciences, Bhopal, Madhya Pradesh, India; 7 Department of Microbiology, Kasturba Medical College, Manipal, Manipal Academy of Higher Education, Manipal, Karnataka, India; 8 Manipal Institute of Virology, Manipal Academy of Higher Education, Manipal, Karnataka, India; 9 Department of Respiratory Medicine, Kasturba Medical College, Manipal, Manipal Academy of Higher Education, Manipal, Karnataka, India; Manipal Academy of Higher Education School of Life Sciences, INDIA

## Abstract

**Background:**

Pulmonary tuberculosis, caused by the bacillus *Mycobacterium tuberculosis*, remains a major global health challenge, particularly in developing countries. In this study, we analyzed publicly available 16S amplicon sequencing datasets from four geographical locations using a single workflow.

**Methods:**

We employed Quantitative Insights Into Microbial Ecology v.2 for microbial diversity analysis and Phylogenetic Investigation of Communities by Reconstruction of Unobserved States v.2 for functional pathway predictions of the gut microbiota in patients with PTB and antitubercular therapy.

**Results:**

Our analysis revealed statistically significant alpha diversity differences in West Africa with decreased microbial diversity in pulmonary tuberculosis patients after two months of antitubercular therapy. Additionally, there were no statistically significant differences observed in pairwise comparisons within the same location or in the aggregate beta diversity of the datasets. The predicted microbial metabolic pathways related to vitamin biosynthesis, amino acid synthesis, and energy production were depleted in pulmonary tuberculosis patients following antitubercular therapy.

**Conclusions:**

The observed alterations of gut microbial diversity and predicted functional profile underscores the influence of antitubercular therapy on gut health, suggesting that longer treatment durations may aggravate these alterations in gut microbial function. Moreover, geographical location exerts a more significant impact on microbial diversity than the disease state in a specific location, highlighting the potential for precision medicine to tailor interventions based on individual or regional microbiome characteristics.

## Introduction

Pulmonary tuberculosis (PTB), caused by the bacillus *Mycobacterium tuberculosis*, remains a major global health challenge, particularly in developing countries [1]. High throughput sequencing enabled the rapid collection of large amounts of information pertaining to microbiomes [2]. Among the various sequencing techniques, the development of amplicon sequencing of the 16S rRNA gene in prokaryotes has opened new frontiers in microbial community analysis [3]. This technique offers a cost-efficient approach to identifying the microbial phylotypes present in samples [4]. While the pathogenesis of tuberculosis has been extensively studied, the potential role of gut microbiota in modulating disease susceptibility and progression is not yet fully understood [5]. However, the use of amplicon sequencing methods has accelerated the bacterial profiling of gut microbiota in tuberculosis disease [6].

To date, numerous methodologies have been devised for the processing of 16S rRNA gene amplicon sequencing data [7]. It is known that the use of different bioinformatic pipelines, workflow, and reference databases for taxonomic assignment can have an impact on the determined microbiota composition [8–10]. Studies focusing on the gut microbiota in PTB patients using 16S amplicon sequencing employed various bioinformatics workflows [6,11]. So far, there is no single gold standard pipeline and/or workflow for 16S amplicon sequencing data, which means that different tools and different parameters for the same step are being used in different pipelines [10].

In the present study, we analyzed publicly available 16S amplicon sequencing data from four geographical locations using a single workflow to study the characteristics of the gut microbiota in patients with PTB.

## Methods

### Datasets search

We have searched systematically in electronic databases such as PubMed, Scopus, Embase, and Web of Science using the keywords “gut microbiome”, “gut microbiota”, and “pulmonary tuberculosis” using Boolean operators like “AND” and “OR”. We have also searched in Genome online databases and National Center for Biotechnology Information (NCBI) sequenced read archives (SRA). We found 17 studies on tuberculosis and gut microbiota. The screening process used to select the final datasets is outlined in S1 Fig in Supporting information File 1. Out of 17 studies, we excluded 10 studies since those studies did not upload any raw sequences. In the remaining seven studies, we performed a screening to select final datasets based on metadata availability. Of these, three studies were excluded: one due to the absence of 16S amplicon data and two because the group information could not be determined from the SRA run info table. Finally, four datasets comprising 213 16S amplicon sequences were selected (S1 Fig in Supporting information File 1).

### Criteria for dataset screening and selection

We considered the datasets with PTB patients’ group with a comparator group consisting of individuals without PTB, such as healthy household contacts (HHC), healthy controls (HC), or close contacts (CC). We did not merge all comparator groups, as the definition of “healthy control” varied across the four datasets, which originated from four different locations (India (I), Taiwan (T), West Africa (W), and South Africa (S)). Maintaining this distinction ensured that differences in control group definitions did not confound our analysis. We also considered PTB patient groups with antitubercular therapy (ATT) exposure (PTB patients at one week of antitubercular therapy, TBW; PTB patients at one month of antitubercular therapy, TBM; and PTB patients at two months of antitubercular therapy, TB2M).

### Selection of datasets and retrieval

We performed initial data screening and selection steps for the required raw reads. First, we downloaded the ‘runinfo’ table from the NCBI SRA database, which contains metadata information for sequence runs that are available in the repository. Subsequently, we used the metadata from the ‘runinfo’ table to identify the required SRA IDs. In cases where the groups could not be identified using this metadata, we contacted the corresponding study author for further information. Subsequently, we identified and screened the required SRA IDs based on predefined inclusion criteria. The screening process involved group types. Four datasets were selected for our analysis (Table 1). Upon finalizing the list of targeted SRA IDs from all four selected datasets, to retrieve the corresponding raw sequenced reads from the NCBI SRA, we utilized the SRA Toolkit version 3.0.10 installed on a Linux operating system.

**Table 1:** Overview of selected datasets.

Datasets (Study) [ref]	Bio project ID/ SRP Accession	Region, Country (City)	Hypervariable region	PTB group	Comparator group
Dataset 1 (Maji et al. 2018) [12]	SRP079687	South Asia, India (New Delhi)	V3	TB = 6 TBW = 6 TBM = 6	HHC = 6
Dataset 2 (Huang et al. 2019) [13]	PRJEB29035	East Asia, Taiwan (Taipei)	V3-V5	TB = 31	HC = 20
Dataset 3 (Namasivayam et al. 2020) [14]	PRJNA622267	West Africa, Mali	V4	TB = 20 TB2M = 20	HC = 10
Dataset 4 (Naidoo et al. 2021) [15]	PRJNA664352	South Africa (Cape town)	V4	TB = 31	CC = 57

### Preprocessing and quality control

The obtained raw reads for the respective datasets were assessed for quality using FastQC (version: fastqc-0.12.1). Subsequently, we employed BBDuk (version: bbmap-39.06–0), which utilized a stringent trimming algorithm to remove adapter sequences, low-quality bases, and primer sequences, thereby enhancing the overall quality of the data. After obtaining the trimmed reads, we conducted a second round of FastQC to evaluate the impact of the trimming process on the sequencing data.

### Amplicon sequence variant (ASV) generation and taxonomic classification

The trimmed reads obtained after the preprocessing and quality control were then imported into Quantitative Insights Into Microbial Ecology (QIIME2) (Version: QIIME2–2023.5) [16]. We have used the Divisive Amplicon Denoising Algorithm 2 (DADA2) plugin within QIIME2 for denoising and clustering of amplicon sequence variants (ASVs). Alpha-rarefaction plot at a sampling depth of 12,400, covered all the samples. Taxonomic classification of the ASVs was performed using the q2-feature-classifier plugin. We have used a 16S rRNA full-length classifier trained on the SILVA v138 database [17] to assign taxonomic labels.

### Statistical analysis

The downstream statistical analyses were conducted in R using packages such as phyloseq v1.48.0 [18], microbiome v1.26.0 [19] and microeco v1.12.0 [20]. These packages include useful functions to manage and subset the ASV abundance tables along with the option to add ggplot2 layers for the generated plots. Alpha diversity metrics, including observed species and Shannon diversity index, were calculated. Beta diversity analysis was conducted to assess the microbial community composition between samples using weighted UniFrac distances. The pair-wise significant differences for alpha diversity among groups within the locations were assessed using the Wilcoxon Rank Sum test (overall significance was calculated using Kruskal-Walli’s test). Beta diversity was analyzed using weighted UniFrac metrics and visualized with Principal Coordinate Analysis (PCoA). The compositional variations among the groups were found to be statistically significant based on PERMANOVA test. We have run the linear discriminate analysis (LDA) with effect size (LEfSe) measurement to identify differentially abundant taxa in pairwise comparison [21], which gives an overview of the discriminant taxa in this rarefied dataset. LEfSe implementation of microeco package, which includes p-value correction by False Discovery Rate (FDR) method, was used to identify discriminant taxa. However, after p-value correction, significant differences were detected only in the sub-groups of the West African population.

The QIIME2 output files and the R script may be accessed on Zenodo at https://doi.org/10.5281/zenodo.15321064, for reproducing the results.

### Predicted metabolic gene pathway analysis

The functional potential of microbial communities was inferred using the Phylogenetic Investigation of Communities by Reconstruction of Unobserved States (PICRUSt2) plugin (Version: PICRUSt2-2.5.2) [22]. The obtained predicted MetaCyc abundances (unstratified pathway abundances) were analyzed with Statistical Analysis of Metagenomics Profiles (STAMP) [23,24]. We used STAMP to assess the significance of differences in microbial functional profile predictions in groupwise comparisons within the dataset, and differences were analyzed using Welch’s t-test option. Results were considered statistically significant when p < 0.05.

### Ethics approval

The study used publicly available 16S amplicon sequenced data from NCBI SRA, and no ethical approval was sought.

## Results

### Alpha diversity analysis

**Fig 1:**
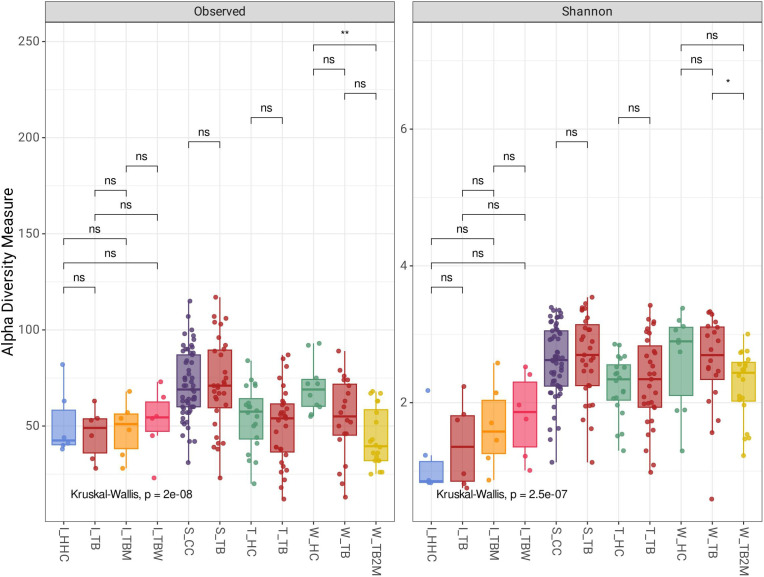
Alpha diversity. Alpha diversity illustrates the Observed taxa and Shannon index among the groups.

Analysis of the alpha diversity box plot for the Shannon index and observed taxa (Fig 1) showed differences among groups within the locations, indicating that the microbial community of gut microbiota changed with PTB disease and/or with exposure to the ATT. However, these were not statistically significant, except for the West African population (Dataset 3), where we observed statistically significant differences (p < 0.001) (Fig 1) for observed taxa between healthy individuals and PTB patients at two months of ATT. In the West African population, we also observed statistically significant differences (p < 0.05) (Fig 1) in the Shannon index between PTB patients before ATT and PTB patients at two months of ATT.

### Beta diversity analysis

**Fig 2:**
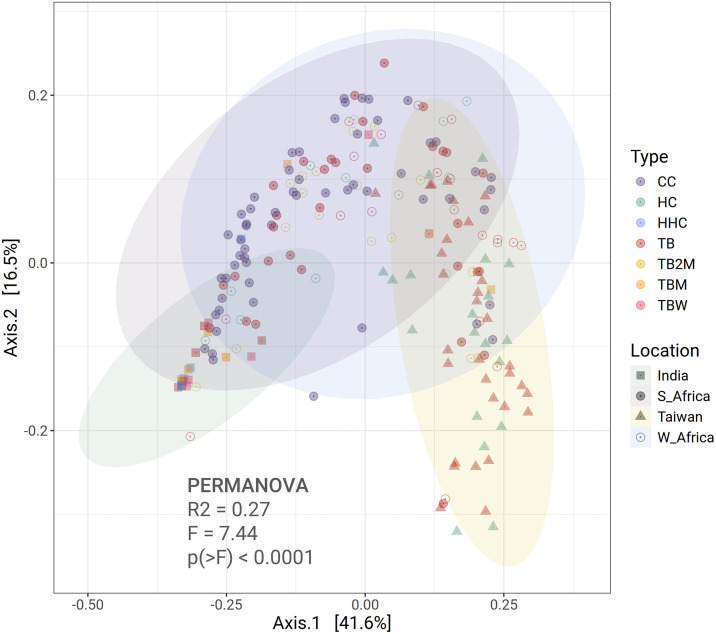
Beta Diversity. Beta diversity illustrates the weighted UniFrac distances among the groups.

We observed a notable difference among groups across the four locations in the PCoA plot based on weighted UniFrac distances (Fig 2). Several statistically significant pairwise comparisons between different locations were found, including comparisons as India (I_HHC) vs. Taiwan (T_HC, T_TB), India (I_HHC) vs. West Africa (W_TB, W_TB2M), India (I_HHC) vs. South Africa (S_CC, S_TB), Taiwan (T_TB) vs. West Africa (W_HC, W_TB2M), Taiwan (T_HC) vs. West Africa (W_HC), Taiwan (T_HC) vs. South Africa (S_HC, S_CC), Taiwan (T_HC) vs. India (I_TBW, I_TBM), Taiwan (T_TB) vs. South Africa (S_HC, S_CC), and Taiwan (T_TB) vs. India (I_TB, I_TBW) (Fig 2). However, no statistically significant differences were found in pairwise groups from the same location. Additionally, no significant differences were observed in overall beta diversity across the datasets. This outcome suggests that geographical location plays a more integral role in shaping microbial diversity than disease state within a location.

### Discriminant taxa

**Fig 3:**
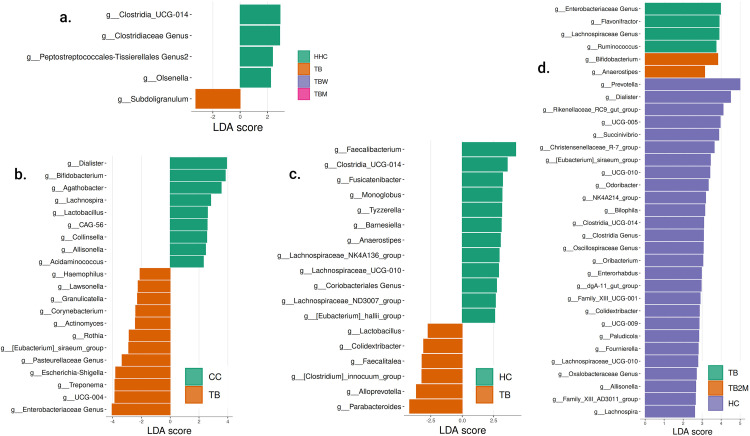
LEfSe Analysis. LEfSe Analysis illustrates a LDA score greater than 2 (p < 0.05) of genera among the groups in the followings: **a.** Indian Population; **b.** South African Population; **c.** Taiwan Population; **d.** West African population.

From the pairwise Linear discriminate analysis effect size (LEfSe) comparisons, we have discovered marker taxa at the genus level in four datasets (Fig 3) with unadjusted p-values. The highest number of discriminating genera was identified in the African population (West and South Africa), followed by the Asian population (India and Taiwan). In the Indian population, we found the genus *Subdoligrnulum* to be abundant in the TB group, whereas *Peptostreptococcales-Tissierellales, Olsenella,* and *Clostridia_UCG-014* genera were differentially abundant in the HHC group. The genus *Clostridia_UCG_014* was identified in healthy individual groups (HHC, HC) of the Indian, Taiwan, and West African populations.

In the Taiwan population, 18 discriminative genera were identified, among which 12 belong to HC and 6 to the TB group. Some of the unique marker genera in the HC group were *Faecalibacterium, Monoglobus, Fusicatenibacter, Tyzzerella, Barnesiella, Lachnospiraceae_NK4A136_group, Coriobacteriales,* and *[Eubacterium]_hallii_group*. Whereas the unique marker genera in TB group were identified as *Lactobacillus, Colidextribacter, Faecalitalea, [Clostridium]_innocuum_group, Alloprevotella,* and *Prabacteroides.* The genera *Colidextribacter* and *Lactobacillus* were also identified in the HC group of West Africa and the CC group of South Africa, respectively.

A total of 35 discriminative genera were found in the West African population, of which 27 are in the HC group, six are in the TB group, and two are in the TB2M group. Some of the unique genera markers in the HC group were *Prevotella, Rikenellaceae_RC9_gut_group, Succinivibrio, Christensenellaceae_R_7_group, [Eubacterium)_siraeum_group, Odoribacter,* NK4A214_group*, Bliophila, Oscillospiraceae genus, Oribacterium, Enterorhabdus,* dgA-II_gut_group*,* Famliy_XII_UCG_001*, Paludicola, Fournierella, Oxalobacteraceae,* and *Allisonella,* Family_XIII_A23011_group*.* In the TB group, the marker genera were identified to be the *Lachnospiraceae genus, Flavonifractor, Ruminococcus, Anaeroplasma,* and *Izemoplasmatales.* The taxa *Enterobacteriaceae genus* was found in the TB group of the African population (West and South Africa). The genus *Anaerostipes* was identified in both the TB2M group of West Africa and the HC group of the Taiwan population. The genus *Bifidobacterium* was identified in the TB2M group of West Africa and the CC group of the South African population.

9 of the 21 discriminative genera found in the South African population are in the CC group, and the remaining 12 are in the TB group. Some of the marker genera in the CC group were identified to be *Agathobacter, CAG-56, Collinsella, Allisonella,* and *Acidaminococcus.* The marker genera in the TB group were *Haemophilus, Lacusonella, Granulicatella, Corynebacterium, Actinomyces, Rothia, Pasteurellaceae genus, Escherichia-Shigella,* and *Treponema.* The identified genus *[Eubacterium]_siraeum_group* in the TB group of the South African population was also identified in the West African population HC group. Genera *Dialister* and *Lachnospira* were found in healthy individuals (HC and CC group) of both the West and South African populations.

### Dominant taxa

**Fig 4:**
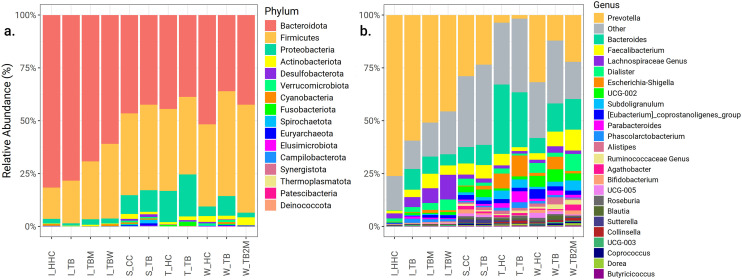
Relative abundance. Relative abundance of bacteria illustrated among the groups in the following taxa levels: **a.** At Phylum level; **b.** At genus level.

Based on relative abundance, at the phylum level, we observed *Bacteroidota, Firmicutes, Proteobacteria,* and *Actinobacteriota* were predominant across India, Taiwan, West and South Africa (Fig 4). The phyla *Proteobacteria and Actinobacteriota* were more abundant in Taiwan, South Africa, and West Africa than in India. At the genus level, Prevotella was observed to be the least abundant in the Taiwan population. Genus *Escherichia-Shigella* was relatively more abundant in the Taiwan population than in the West and South African populations (Fig 4).

**Fig 5:**
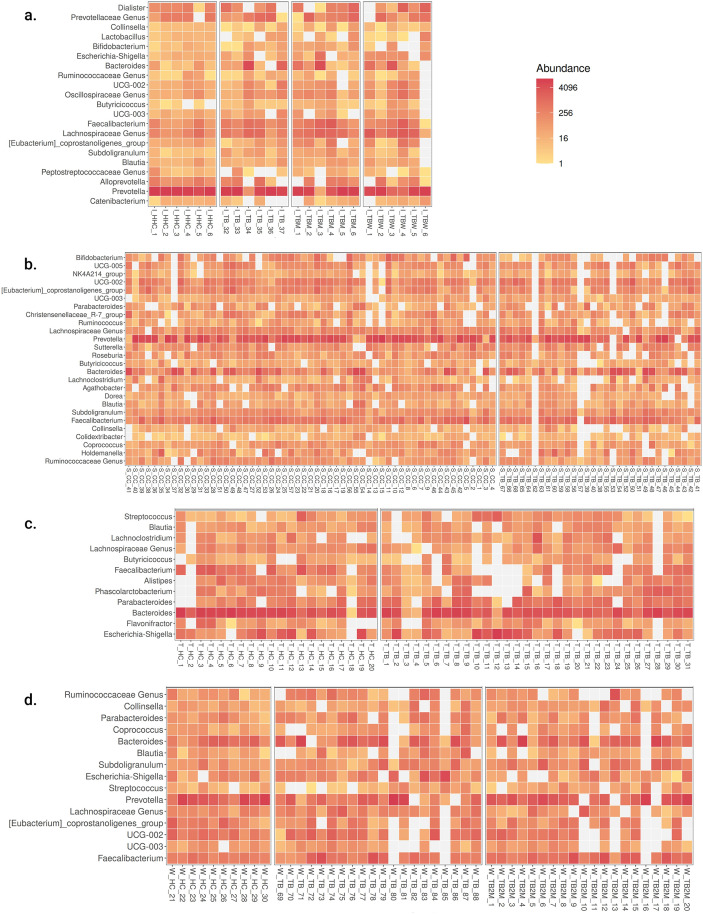
Heatmap of topmost bacteria genera. The heatmap of topmost genera among the groups in followings: **a.** Indian Population; **b.** South African Population; **c.** Taiwan Population; **d.** West African population.

The heatmap (Fig 5) illustrates the topmost bacterial genera across the study groups in all four geographical locations. Irrespective of the presence of TB disease or its treatment exposure, we found that *Blautia, Bacteroides,* and *Faecalibacterium* were the most prominent bacterial genera in all four locations (India, Taiwan, West and South Africa). We found Prevotella to be a prominent genus in India, West, and South Africa. Specifically, *Dialister, Lactobacillus,* and *Alloprevotella* were found as prevalent genera in India, while Alistipes, Phascolarctobacterium, and Flavonifractor were more common in the Taiwan population. The genus *Coprococcus* was frequently observed in both West and South African populations. In contrast, the genera *Holdemanella, Colidextribacter, Dorea, Agathobacter, Roseburia, Sutterella, Ruminococcus, Christensenellaceae_R_7_group,* and *NK4A214_group* were particularly notable in the South African population.

### Prediction of the functional potential of metabolic pathways

The PICRUSt2 analysis revealed prediction of several significant differences among groups across all datasets (S2-S11 Fig in Supporting information File 1). Table 2 describes the number of predicted active features identified in groupwise comparison. Detailed descriptions of the predicted abundant metabolic pathways are provided in Supporting information Files 2–5.

**Table 2:** Number of active features in group-wise comparison.

S.N.	Datasets [Ref]	Groupwise comparison	Number of active features*
1	Dataset 1 [12]	HHC Vs. TB	12
		HHC Vs. TBW	27
		HHC Vs. TBM	18
		TB Vs. TBW	0
		TB Vs. TBM	1
		TBW Vs. TBM	1
2	Dataset 2 [13]	HC Vs. TB	13
3	Dataset 3 [14]	HC Vs. TB	46
		HC Vs. TB2M	78
		TB Vs. TB2M	94
4	Dataset 4 [15]	CC Vs. TB	48

*The number of predicted active features represents the number of the predicted metabolic pathways abundance between groups (P < 0.05).

In a group-wise comparison from the Indian population, we predicted that the PWY-6263 (superpathway of menaquinol-8 biosynthesis II) declined in all groups (i.e., TB, TBW, and TBM) compared to HHC (S2-S4 Fig in Supporting information File 1). In Taiwan population, the predicted pathway ANAEROFRUCAT-PWY (homolactic fermentation), ASPASN-PWY (superpathway of L-aspartate and L-asparagine biosynthesis), PWY-7199 (pyrimidine deoxyribonucleosides salvage), PWY0-1261 (peptidoglycan recycling I), and PWY-7371 (5,8-dihydroxy-2-naphthoate biosynthesis II) were inferred to be reduced in TB group (S7 Fig in Supporting information File 1). In the West African population, the group-wise comparison revealed a decline of inferred THISYN-PWY (super pathway of thiamine diphosphate biosynthesis I), ARG+POLYAMINE-SYN (super pathway of arginine and polyamine biosynthesis), TRPSYN-PWY (L-tryptophan biosynthesis), and P23-PWY (reductive TCA cycle I) pathways in the TB group (S8 Fig in Supporting information File 1). We predicted that the common pathway TRPSYN (L-tryptophan biosynthesis) was inferred to be depleted in the TB group in both West and South Africa (S8 & S11 Fig in Supporting information File 1).

To evaluate the impact of ATT on predicted metabolic pathways of gut microbiota of PTB patients, we conducted a group-wise comparison with and without ATT in Indian and West African populations (S2-S6, S8-S10 Fig in Supporting information File 1). In the Indian population, no predicted significant differences were observed after one week of therapy (i.e., TB vs. TBW group). However, we inferred that the PWY-6944 (androstenedione degradation I (aerobic)) pathway was predicted to be depleted in the TBM group compared to the TB group. In contrast, the PWY-7209 (superpathway of pyrimidine ribonucleosides degradation) pathway was inferred to be depleted in the TBM group compared to the TBW group (S2-S6 Fig in Supporting information File 1). Interestingly, in the West African population, we found 62 predicted metabolic pathways depleted after two months of ATT compared to PTB patients before the initiation of ATT (S8-S10 Fig in Supporting information File 1).

## Discussion

Our study is the first of a kind where we employed a single workflow to study the microbial communities and predicted potential functionalities of gut microbiota in PTB patients from diverse populations. By analyzing publicly available 16S amplicon-sequenced data from four geographically diverse locations, we observed regional differences in the taxonomic makeup and predicted functional pathways of the gut microbiota.

Alpha diversity is a quantitative measure that summarizes the structure of a microbial community within a single metagenomic sample, focusing on either richness, evenness, or both. Richness refers to the number of distinct species present in the sample. At the same time, evenness describes how uniformly those species are distributed in abundance [25,26]. Our findings from alpha diversity analysis revealed a statistically significant difference in microbial richness (Observed taxa) between healthy individuals and PTB patients after two months of ATT in the 16S amplicon sequenced data from West Africa, which suggests that specific microbial taxa at the species level are substantially depleted in PTB patients following two months of ATT compared to a healthy population. In the same West African population, PTB patients before ATT initiation and PTB patients after two months of ATT displayed a significant difference in microbial evenness (Shannon index). This finding indicates that while the richness may not differ significantly, the distribution of taxa (evenness) changes considerably throughout therapy. Alpha diversity changes suggest a notable alteration in the balance of microbial populations as treatment progresses, potentially signifying therapeutic impacts or the emergence or depletion of specific taxa during treatment.

The depletion in gut microbial richness and evenness may have significant implications for patient health, as alterations in gut microbiota have been associated with immune function and overall well-being [27]. Further research is necessary to investigate whether these changes in microbial diversity during ATT influence treatment outcomes or the recovery of patients. Only the findings from the West African population showed statistically significant changes in richness and evenness. The trend in the Taiwan population indicates that PTB disease could alter gut microbial diversity (in terms of richness and evenness), but it was not statistically significant.

Interestingly, the alpha diversity analysis from the Indian population showed a different pattern where the microbial richness and evenness increased with PTB disease followed by ATT. TB disease and ATT are associated with decreased gut microbiota diversity, as observed in other studies [28–30]. Moreover, evidence suggests increased gut microbiota diversity with the presence of TB disease as well ATT [21–33]. The richness in gut microbial diversity is considered to have shielding effects on inflammatory diseases [34]. Antimicrobial agents are responsible for the depletion of gut commensal microorganisms and altering gut microbiota composition. Therefore, the altered gut flora of TB patients may be a signal for weakened anti-inflammatory ability [28]. Studies showed that gut microbiota-influenced anti-inflammatory conditions were related to increased bacterial diversity in TB patients, including many opportunistic pathogens [12,35]. Therefore, it is essential to consider that TB disease and ATT-induced disturbances in the gut microbial community could create conditions that facilitate the proliferation of pathobionts [36]. A similar trend was observed in the South African population, where richness and evenness were higher in healthy individuals (CC) than in the TB group; however, these differences did not reach statistical significance.

In our study, beta diversity was evaluated using the UniFrac distance metric, which is particularly useful in investigating whether evolutionary lineages of bacteria are shared or distinct across groups, offering a phylogenetic view of community composition [37]. Unweighted UniFrac gives phylogenetic relationships between microbial communities and highlights differences based on the presence or absence of taxa. On the other hand, weighted UniFrac gives the phylogenetic relationships between microbial communities and highlights differences based on their relative abundance [37].

Our study findings reveal a significant difference in the weighted UniFrac distance between microbial communities in pairwise group comparisons between different locations (India, Taiwan, West Africa, and South Africa). This indicates a phylogenetic shift in gut microbiota, where specific evolutionary bacterial lineages are either lost or newly acquired in response to TB and its treatment, highlighting the more profound evolutionary changes in the bacterial community caused by TB and ATT. However, the lack of overall beta diversity differences across the entire dataset and the lack of statistically significant pairwise differences among groups within the same geographic location highlight how important geographic location is in determining gut microbial diversity. These findings imply that regional pathogen exposure, socioeconomic circumstances, cultural practices, and diet may impact microbiota composition more than disease state alone. Although prior studies [11,28–33,35] frequently emphasize disease-specific alterations in microbiota, the geographical variation noted here indicates that region-specific factors may influence the impact of TB and ATT on microbial diversity. The observed influence of geographical location on the gut microbiome likely reflects a composite effect of multiple intertwined factors rather than geography alone. Dietary habits, which vary substantially across regions, represent one of the most significant determinants of gut microbial structure and function. For example, populations consuming fiber-rich, plant-based diets typically harbor higher abundances of short-chain fatty acid producing bacteria, whereas high-fat or protein-heavy diets are associated with distinct compositional signatures [38]. Beyond diet, host genetic background, cultural lifestyle practices, sanitation infrastructure, and environmental microbial exposures contribute collectively to shaping local microbiota ecosystems. A recent study demonstrated that residential environmental factors, including the indoor microbiome and metabolite environment, significantly influence the composition and diversity of human gut microbiota [39]. Likewise, lifestyle and behavioral factors such as physical activity, stress, and urbanization have been shown to modulate the gut microbial ecosystem, interacting with dietary and environmental influences to produce region-specific community structures [40]. These findings underscore that the “geographical effect” observed in our analysis likely represents an integrated signature of diet, environment, and host factors that together modulate the gut microbial landscape across populations. It may elucidate why microbiota alterations linked to TB and ATT, observed in one locale, may not be consistently replicated in another.

Marker taxa detection helps identify microbial signatures that differentiate in a comparative investigation. In 16S-based phylogenies, LEfSe provides biomarkers that explain abundant differentiating phenotypes or taxa, highlighting which microorganisms are associated with specific conditions. The visualizations from LEfSe map the identified biomarkers onto taxonomic trees, summarizing the results in a biologically meaningful way, as this statistically and visually captures the hierarchical relationships [21]. In our analysis, the ability of LEfSe to detect subtle differences in microbial composition between groups within a location was paramount. The results highlight distinct microbiota community marker taxon patterns in all four populations, with the highest number of discriminating genera identified in African populations. *Clostridia*_*UCG-014* has been linked to various health protective factors, including immunomodulation and gut barrier function [41,42]. *Clostridia_UCG-014* was the marker taxon in healthy individuals across multiple populations (India, Taiwan, and West Africa) in our study, which implies protective microbiome characteristics that may confer resistance to TB infection.

The *Enterobacteriaceae* family is known to comprise both commensals and pathobionts. *Enterobacteriaceae* usually constitutes a small fraction of the healthy gut microbiota. This family includes a diversity of bacterial genera, including *Escherichia*, *Enterobacter*, and *Shigella* [43,44]. The species *Escherichia coli* was shown to be, by far, the most dominant *Enterobacteriaceae* in healthy humans. However, other genera of *Escherichia* and *Shigella* are associated with pathological conditions [45]. *Escherichia/Shigella* enterotypes dominate the gut microbiota in patients with acute gastroenteritis and are predicted to have more pro-inflammatory characteristics than enterotypes dominated by *Bacteroides* or *Faecalibacterium*. This suggests that when *Escherichia/Shigella* becomes dominant, it may contribute to inflammation and worsening symptoms in gastroenteritis patients [46]. Furthermore, in another study on the Asian population, *Escherichia/Shigella* genera were found to be dominant in patients with TB meningitis while correlating with elevated plasma tumor necrosis factor-alpha (TNF-alpha) [47]. TNF-alpha is the cytokinin that plays an important role in TB pathogenesis [48]. In the present study, we identified the genera *Escherichia/Shigella* under the *Enterobacteriaceae* family as relatively abundant in Taiwan, West African, and South African populations. Interestingly, within the South African population, we identified *Escherichia/Shigella* as a marker taxon in PTB patients.

It is important to note that the control categories used across the included studies (HHC, HC, and CC) represent distinct exposure contexts. While HC are typically community participants without known exposure to TB, HHC and CC often share the same living environment as PTB patients and may have subclinical or latent *Mycobacterium tuberculosis* infection. Prior studies have suggested that such individuals can exhibit mild immune activation, systemic inflammation, or even modest alterations in gut microbiota composition compared to unexposed healthy individuals [49,50]. This potential intermediate immunological and microbial state might have attenuated the distinction between “healthy” and “disease” groups, thereby partially influencing the observed effect sizes in our comparisons. Nonetheless, analyzing these groups separately allowed us to preserve the biological and epidemiological distinctions among the cohorts.

Our findings of the predicted functional profile of metabolic pathways for the gut microbiota of PTB patients indicated several microbial metabolic pathways were inferred to be significantly depleted, which might impact PTB patients’ health as well as the progression of the disease. In all four datasets, we consistently observed predicted depletion in pathways related to vitamin biosynthesis, amino acid synthesis, energy production, and stability of cell walls, highlighting the extensive effects of TB and its treatment on the function of gut microbes. Interestingly, in the Indian population, we inferred the depletion of the PWY-6263 pathway (super pathway of menaquinol-8 biosynthesis II) in all groups compared to healthy individuals. Menaquinones are also known as vitamin K_2_, which are essential, mostly for the posttranslational modification of certain proteins that are required for blood coagulation. Humans cannot synthesize menaquinones; instead, they usually receive a sufficient amount from diet and intestinal bacteria [51]. The deficiency of menaquinones leads to several complications, such as hemorrhage, bone disorder, increased cardiovascular disease risk, and cognitive decline. The depletion of the inferred super pathway of menaquinol-8 biosynthesis II across TB, TBW, and TBM groups in the Indian population (dataset 1) may indicate a shared metabolic impairment due to TB, irrespective of treatment status. From our predicted functional analysis, we inferred that the duration of ATT plays a critical role in modulating microbial metabolic activities. In the Indian population (dataset 1), no significant predicted microbial metabolic pathway alteration was observed after one week of ATT. One predicted depleted pathway was observed after one month of ATT in the same dataset. However, the West African population (dataset 3) was predicted to exhibit a substantial depletion of 62 microbial metabolic pathways after two months of ATT. These findings suggest that prolonged ATT induces a more prominent disruption in the predicted metabolic potential of the gut microbiota.

The enterotypes study by Mobeen et al. 2018 identified *Prevotella* and *Bacteroides* as dominant in the Asian and African continents, which is consistent with our study findings [52]. However, unlike Mobeen et al. 2018 [52], we found *Bifidobacterium* in Asia and Africa. These differences could be attributed to the data from Mobeen et al. 2018 [52], where samples were exclusively from healthy individuals. On the other hand, our dataset includes HHC and/or CC of PTB patients, who might have varying degrees of exposure to TB and potentially altered immune statuses. The probable exposure might have influenced the overall gut microbiota composition, consequently contributing to the observed differences. However, it is a highly debatable area considering the current insufficient evidence to conclusively determine whether gut microbiota signature changes prior to TB disease progression or because of it [5].

Our study’s finding of the variations in gut microbiota across different geographic regions emphasizes the necessity of implementing a precision therapeutic approach to managing microbiome health in TB patients during ATT. In a precision therapeutic approach, interventions may be customized to the local context and the microbiome characteristics of an individual or population, for example, next-generation probiotics, prebiotics, etc. Next-generation probiotics are “live microorganisms identified based on comparative microbiota analyses that, when administered in adequate amounts, confer a health benefit on the host” [53]. Conventional probiotics are like “One-Size-Fits-All” and are not tailored to specific individual or population characteristics. These traditional probiotics formulations are designed to benefit the overall gut health and be used for common issues like digestive discomfort, immune support, or general microbiome balance. Unlike conventional probiotics, the next generation probiotics present opportunities for personalized probiotic therapies [54]. Therefore, developing personalized probiotic therapies could resolve gut microbiota dysbiosis in specific populations. Precision-based probiotic strategies can enhance patient outcomes and reduce unintended health consequences of treatment by mitigating the deleterious effects of TB disease and ATT on the gut microbiota by considering geographical, dietary, and cultural factors.

### Limitations

We acknowledge a few limitations in this study. The stool sampling methods might have differed across the included datasets and could have contributed to differences in microbiota profiles. Variation in sampling, storage, and processing methods is an intrinsic part of sequencing output. This variability is beyond our control, as we focused on utilizing already available sequenced data. The TB treatment typically lasts six months, the PTB cohort with ATT exposure included in our analysis was limited to a maximum of two months and hence does not capture the full impact of prolonged treatment durations. Our study shows the regional difference in the composition of gut microbiota of PTB patients; we recognize a gap in the scope of this analysis. While collecting the metadata for these four datasets, except for dataset 4 (South Africa), we could not get information on the ethnicities of the collected samples in other datasets (India, Taiwan, and West Africa). Therefore, we performed our analysis based on the geographical collection site of the samples. At the same time, this led to the discovery of regional patterns but limited the exploration of the probable influence of ethnic diversity on the gut microbiota. Addressing this gap in future studies could provide a more in-depth understanding of the influence of ethnicity on gut microbiota composition in PTB patients. Further, the identification of discriminant taxa was based on unadjusted p-values. Our study considered variables such as geographical location and PTB groups with ATT duration in microbial diversity and functional analyses, but other potential variables, including diet, sex, and age, could not be analyzed due to insufficient metadata availability. Future studies with comprehensive metadata will help address these factors more robustly. The datasets have different hypervariable regions (V3, V3-V5, and V4). Different hypervariable regions are known to have inherent biases in their ability to classify bacterial taxa and estimate community diversity. For instance, the V4 region generally provides higher accuracy for identifying members of Firmicutes and Bacteroidetes, while the V3–V5 region can better capture Actinobacteria and Proteobacteria [55,56]. Such methodological heterogeneity might have influenced our findings by masking subtle disease-related differences or amplifying regional patterns due to primer- and region-specific taxonomic biases. Although we applied a standardized downstream bioinformatics workflow to reduce these effects, residual variability due to the use of multiple regions cannot be completely ruled out [55,56]. There are limited studies investigating gut microbiota in PTB patients, especially with publicly available 16S amplicon sequencing data. Further narrowing our inclusion criteria based on the same hypervariable regions would have significantly reduced the number of studies for our analysis. Although variation in targeted hypervariable regions can affect taxonomic resolution, this limitation is inherent in publicly available datasets. It is also important to note that the functional profiling in this study was based on PICRUSt2 predictions derived from 16S rRNA gene data. Such inferences are predictive in nature and may not fully represent the actual functional potential or activity of the gut microbiome. Direct validation using shotgun metagenomic or metatranscriptomic approaches would be required to confirm these predicted functions. Future research with harmonized sequencing protocols is necessary to address this limitation.

## Conclusions

Our comprehensive study of 16S amplicon sequences from various geographical regions demonstrated a substantial reduction in microbial richness and evenness among the West African population after two months of ATT, indicating the influence of ATT disruption on the microbiota’s equilibrium and overall stability. Our findings suggest that geographical location exerts a more substantial impact on shaping the gut microbial community than the disease state within a given geographical location. The lack of significant differences in beta diversity between PTB disease and healthy states suggests that regional factors, such as environment, diet, and sociocultural practices, may dominate the microbiota. Furthermore, we identified prominent marker taxa in populations from India, Taiwan, West Africa, and South Africa through comparative analyses within each region, underscoring ATT’s influence on PTB patients’ gut microbiota. Following taxonomic alterations, we noted a substantial decline in anticipated microbial metabolic pathways associated with vitamin biosynthesis, amino acid synthesis, and energy production in PTB patients after ATT. The significant depletion of metabolic pathways following two months of ATT indicates that extended treatment durations may result in more severe disruptions to the functional capacity of gut microbiota, potentially diminishing the synthesis of essential nutrients, impairing energy metabolism, and causing adverse effects on host health, such as decreased immune function and metabolic imbalances.

## Supporting information

Supporting information File 1 Contains figures S1-S11. (DOCX)

Supporting information Files 2 Detailed descriptions of inferred metabolic pathways for dataset 1. (XLSX)

Supporting information Files 3 Detailed descriptions of inferred metabolic pathways for dataset 2. (XLSX)

Supporting information Files 4 Detailed descriptions of inferred metabolic pathways for dataset 3. (XLSX)

Supporting information Files 5 Detailed descriptions of inferred metabolic pathways for dataset 4. (XLSX)

## Abbreviations

ASVs: amplicon sequence variants
ATT: antitubercular therapy
CC: close contacts of PTB patients
DADA: divisive amplicon denoising algorithm
FDR: false discovery rate
HC: healthy control
HHC: healthy household contacts
I: India
LDA: linear discriminate analysis
LEfSe: Linear discriminate analysis effect size
NCBI: national center for biotechnology information
NMDS: non-metric multidimensional scaling
PICRUSt: phylogenetic investigation of communities by reconstruction of unobserved states
PTB: pulmonary tuberculosis
QIIME: quantitative insights into microbial ecology
S: South Africa
SRA: sequenced read archives
T: Taiwan
TB: PTB patients before ATT initiation
TB2M: PTB patients at two months of antitubercular therapy
TBM: PTB patients at one month of antitubercular therapy
TBW: PTB patients at one week of antitubercular therapy
W: West Africa
