# Supplementary material for: Exploring gut microbiota and its predicted functions in pulmonary tuberculosis: A multi-regional study using public 16S datasets
Source: PLoS One. 2025 Nov 13;20(11):e0336337. doi: 10.1371/journal.pone.0336337 (PMC12614512; doi:10.1371/journal.pone.0336337)
Supplement: Supporting information File 1 — (DOCX) [file pone.0336337.s001.docx]

**Datasets search and screening process**

**Fig S1: Datasets search and screening process**


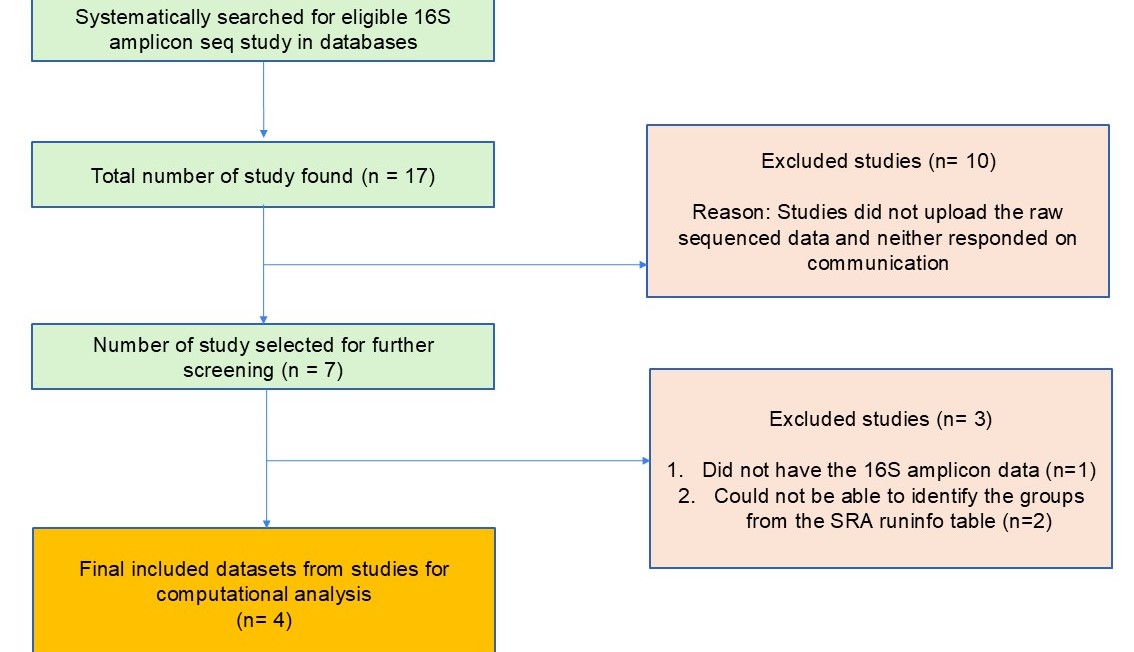


Flowchart illustrates the search and screening process for datasets used in this study.

**Predicted bacterial metabolic pathway alterations among the groups**

**A) Indian Population (Dataset 1)**

**Fig S2: Predicted bacterial metabolic pathway in HHC Vs. TB**


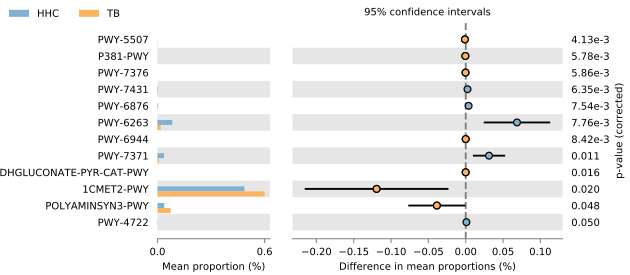


Forest plot illustrates the Predicted bacterial metabolic pathways between HHC & TB in Indian Population. HHC: Healthy households contacts; TB: PTB patients before initiation of antitubercular therapy

**Fig S3: Predicted bacterial metabolic pathway in HHC Vs. TBM**


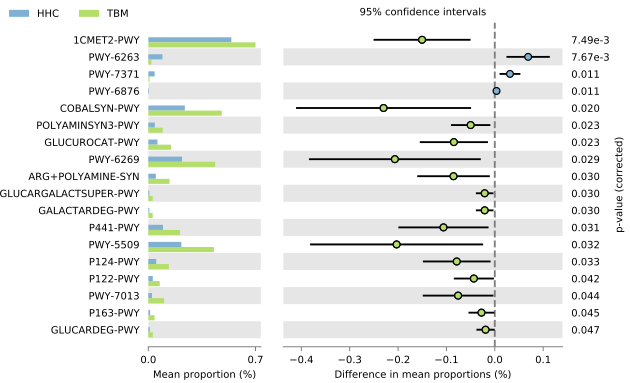


Forest plot illustrates the Predicted bacterial metabolic pathways between HHC & TBM in Indian Population. HHC: Healthy households contacts; TBM: PTB patients at one month of antitubercular therapy

**Fig S4: Predicted bacterial metabolic pathway in HHC Vs. TBW**


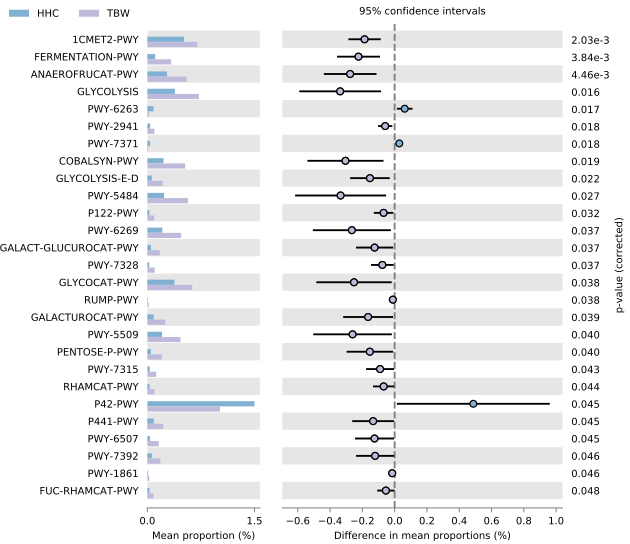


Forest plot illustrates the Predicted bacterial metabolic pathways between HHC & TBW in Indian Population. HHC: Healthy households contacts; TBW: PTB patients at one week of antitubercular therapy

**Fig S5: Predicted bacterial metabolic pathway in TB Vs. TBM**


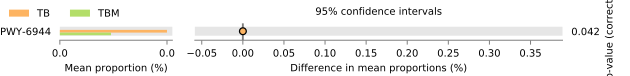


Forest plot illustrates the Predicted bacterial metabolic pathways between TB & TBM in Indian Population. TB: PTB patients before initiation of antitubercular therapy; TBM: PTB patients at one month of antitubercular therapy

**Fig S6: Predicted bacterial metabolic pathway in TBM Vs. TBW**


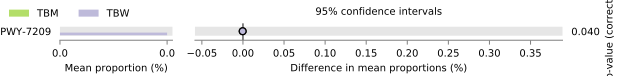


Forest plot illustrates the Predicted bacterial metabolic pathways between TBM & TBW in Indian Population. TBW: PTB patients at one week of antitubercular therapy; TBM: PTB patients at one month of antitubercular therapy

**B) Taiwan Population (Dataset2)**

**Fig S7: Predicted bacterial metabolic pathway in HC Vs. TB**


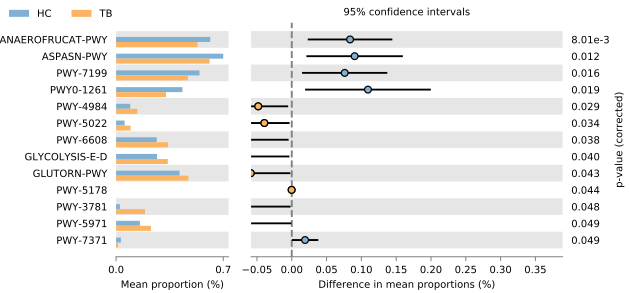


Forest plot illustrates the Predicted bacterial metabolic pathways between HC & TB in Taiwan Population. HC: Healthy control; TB: PTB patients before initiation of antitubercular therapy

**C) West African Population (Dataset 3)**

**Fig S8: Predicted bacterial metabolic pathway in HC Vs. TB**
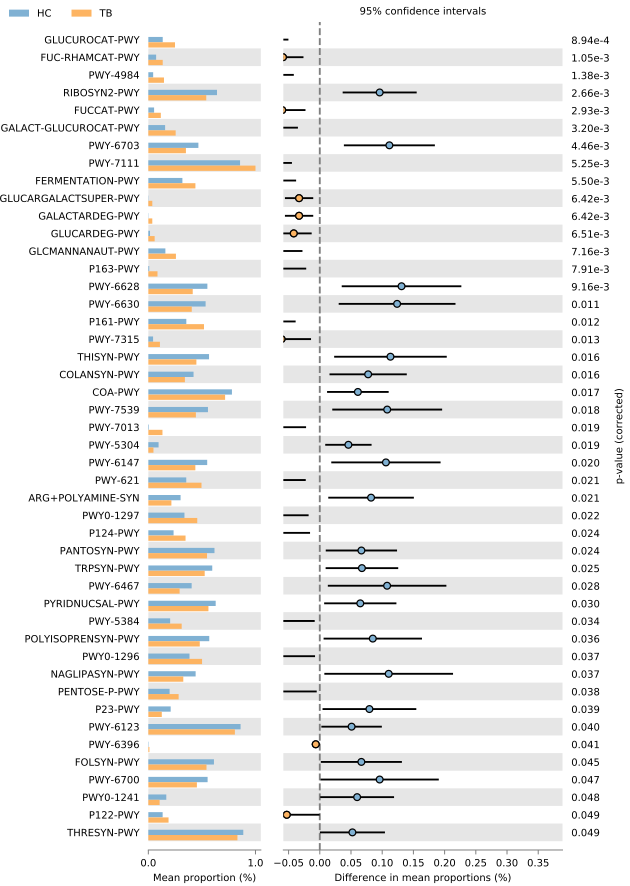


Forest plot illustrates the Predicted bacterial metabolic pathways between HC & TB in West African Population. HC: Healthy control; TB: PTB patients before initiation of antitubercular therapy

**Fig S9: Predicted bacterial metabolic pathway in HC Vs. TB2M**
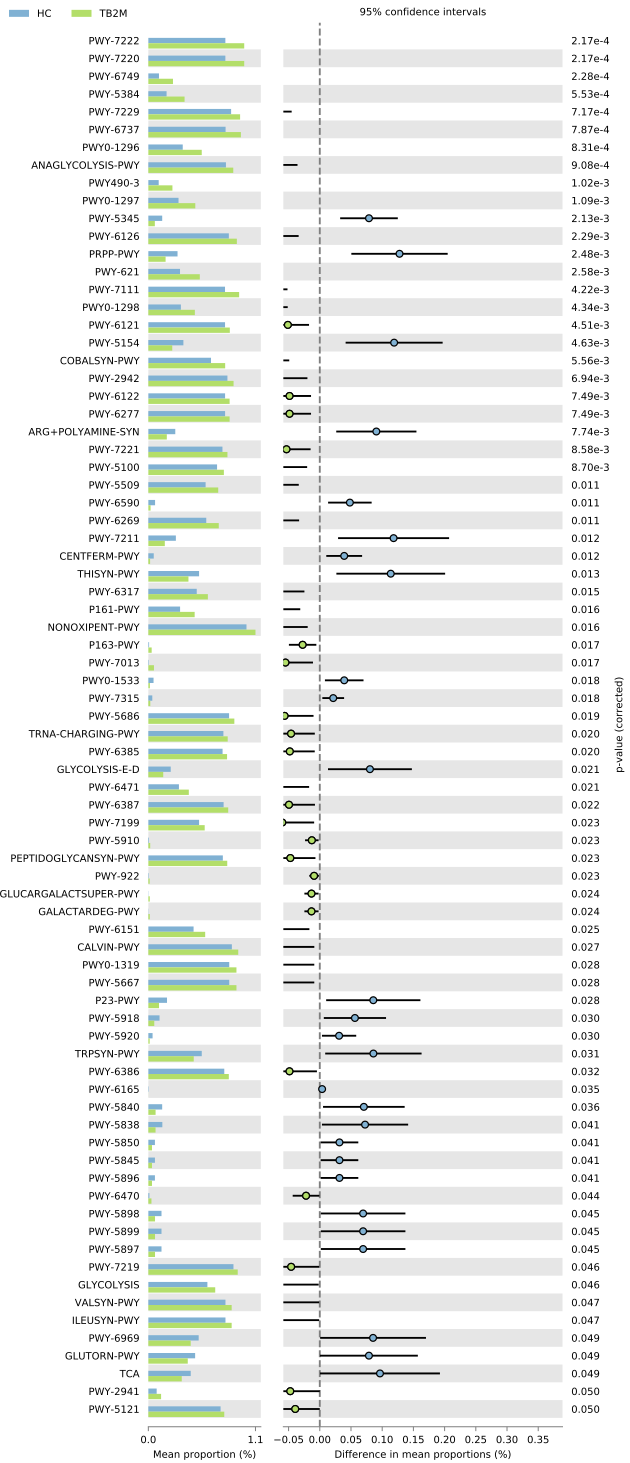


Forest plot illustrates the Predicted bacterial metabolic pathways between HC & TB2M in West African Population. HC: Healthy control; TB2M: PTB patients at two months of antitubercular therapy

**Fig S10: Predicted bacterial metabolic pathway in TB Vs. TB2M**
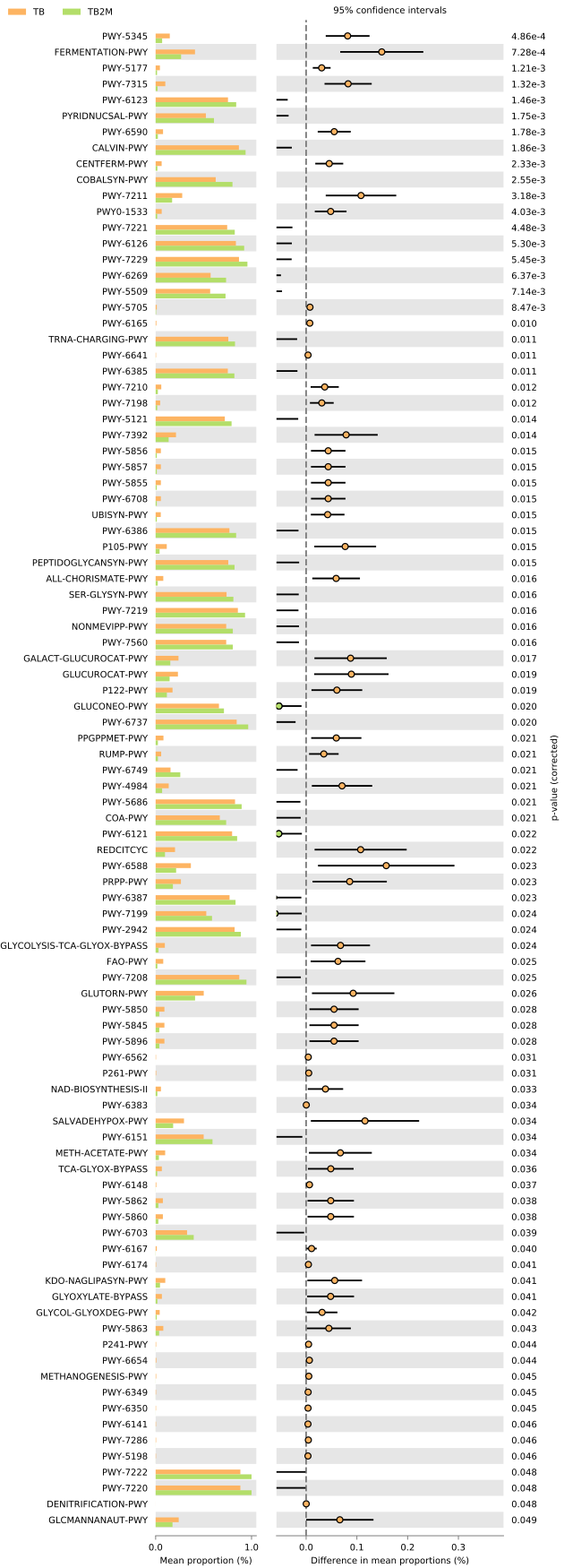


Forest plot illustrates the Predicted bacterial metabolic pathways between TB & TB2M in West African Population. TB: PTB patients before initiation of antitubercular therapy; TB2M: PTB patients at two months of antitubercular therapy

**D) South African Population (Dataset 4)**

**Fig S11: Predicted bacterial metabolic pathway in CC Vs. TB**


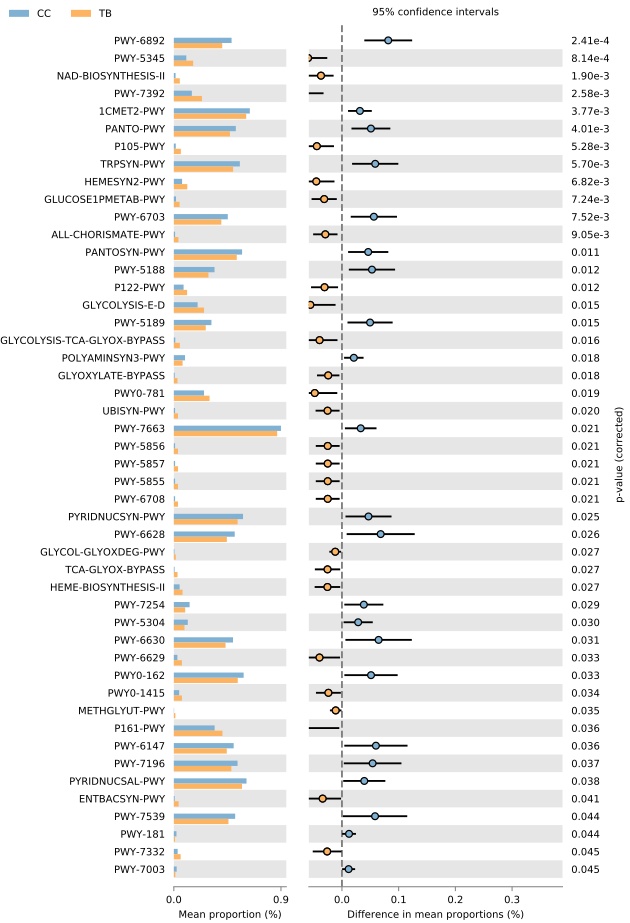


Forest plot illustrates the Predicted bacterial metabolic pathways between CC & TB in South African Population. CC: Close contacts of PTB patients; TB: PTB patients before initiation of antitubercular therapy
